# Intratympanic glucocorticosteroid therapy for idiopathic sudden hearing loss: Meta-analysis of randomized controlled trials

**DOI:** 10.1097/MD.0000000000008955

**Published:** 2017-12-15

**Authors:** Dan Lai, Fei Zhao, Nasim Jalal, Yun Zheng

**Affiliations:** a Hearing Center/Hearing and Speech Science Laboratory, Department of Otolaryngology Head and Neck Surgery, West China Hospital of Sichuan University, Chengdu; b Department of Otolaryngology Head and Neck Surgery, The Affiliated Hospital of Southwest Medical University, Luzhou, China; c Department of Vision and Hearing Sciences, Anglia Ruskin University, Cambridge, England; d Department of Hearing and Speech Science, Xinhua College, Sun Yat-Sen University, Guangzhou, China.

**Keywords:** idiopathic sudden sensorineural hearing loss, initial treatment, intratympanic steroid treatment, meta-analysis, systemic steroids

## Abstract

**Background and objective:**

Glucocorticoids are the standard treatment for idiopathic sudden sensorineural hearing loss (ISSNHL), but whether intratympanic or systemic therapy is superior remains controversial. Previous meta-analyses of this question have omitted key clinical trials or included observational studies.

**Methods:**

English-language randomized controlled trials in OvidSP, PubMed, Embase, CINAHL, and the Cochrane Library comparing intratympanic versus systemic glucocorticoid therapy for ISSNHL were meta-analyzed using RevMan 5.3. The primary outcome of interest was improvement in pure tone average (PTA) threshold.

**Results:**

Six trials involving 248 patients receiving intratympanic steroids and 236 receiving systemic steroids were meta-analyzed. PTA thresholds were similar between the 2 groups at 3 months after therapy initiation (mean difference, 0.24; 95% confidence interval [CI] −2.43 to 2.91, *P* = .86; I^2^ = 54%, *P* = .07, random-effects model). PTA thresholds were also similar at 6 months (mean difference, 4.69, 95% CI −5.84 to 15.22, *P* = .38), although the results showed extremely high heterogeneity (I^2^ = 98%). Sensitivity analysis indicated that a single trial containing 250 patients provided the strongest evidence for equivalence between the 2 types of therapy. Rates of recovery within 3 months (defined as PTA improvement >10 dB) were similar between the 2 types of therapy (odds ratio 0.92, 95% CI 0.59–1.43, *P* = .70), with no significant heterogeneity in the pooled data (I^2^ = 1%, *P* = .40).

**Conclusion:**

Intratympanic and systemic steroids’ therapies appear to show similar short-term efficacy for restoring hearing in patients with ISSNHL. Intratympanic therapy may reduce systemic side effects associated with steroid use.

## 1 Introduction

Idiopathic sudden sensorineural hearing loss (ISSNHL), defined as hearing loss of ≥30 dB in 3 consecutive frequencies of pure tone average (PTA) within 72 h,^[1]^ is estimated to affect 5 to 20 people per 100,000 per year worldwide.^[2]^ The causes of the condition are unknown but are more likely to be a spectrum of pathologies that affect the cochlea, rather than a single pathological change.^[3]^ Primary treatment and interventions for this condition are the subject of ongoing debate, which is made more complex by the fact that 32% to 65% of patients spontaneously recover within 15 days from onset.^[4–7]^ Among the numerous treatments tested,^[8]^ oral/systemic steroid treatment is the most frequent primary treatment and is widely considered the most effective.^[9]^

However, systemic steroids are associated with significant side effects, including mood changes, loss of appetite, disrupted sleep patterns, increased thirst, weight gain, hypertension, and hyperglycemia. Therefore, this therapy should be considered carefully if patients have other chronic diseases, such as diabetes mellitus or glaucoma.^[10]^

An alternative to systemic therapy is intratympanic therapy, first proposed by Silverstein et al in 1996.^[11]^ Local steroid administration directly into the ear reduces the risk of systemic side effects and allows the steroid to penetrate directly into the cochlea and achieve a high concentration there even when low doses are used.^[12,13]^ Steroids administered at the tympanic membrane are usually dexamethasone and solumedrol, which affect immune suppression and ion homeostasis.^[14]^ The principle behind this therapy is that the steroid reduces inflammation associated with labyrinthitis, enhances cochlear blood flow, and improves striavascularis function.^[15]^ In fact, intratympanic therapy can be an effective second-line therapy for patients who do not recover hearing after oral steroid treatment.^[7,10,15–20]^ Despite its advantages, intratympanic steroid therapy is more expensive, requires multiple office visits, and is associated with pain, transient dizziness, infection, and persistent tympanic membrane perforation.^[1]^

The evidence base is unclear on how intratympanic or systemic steroid therapy compare as primary treatments for ISSNHL. Some studies have found the 2 to be equivalent,^[21,22]^ while others have found intratympanic therapy to be superior.^[23–25]^ While 1 meta-analysis^[26]^ concluded that intratympanic therapy is superior to systemic therapy, other meta-analyses have found the 2 types of therapy to be equivalent.^[18,27,28]^ At the same time, some work has shown intratympanic administration to be inferior to combination therapy involving intratympanic dexamethasone and high-dose prednisone taper.^[29]^These discrepancies in the literature prevent definitive conclusions about the optimal primary treatment for ISSNHL.

Since these discrepancies are likely due to inclusion of studies that were not exclusively randomized clinical trials (RCTs) and of studies in which patients were treated with combination therapies, we wished to review and meta-analyze relevant evidence only from RCTs comparing intratympanic and systemic steroid therapy as single therapies, not in combination with other treatments. RCTs can provide the most rigorous assessments to guide clinical practice.^[30]^

## 2 Methods

### 2.1 Search strategy and data sources

The literature search was guided by the following problem formulation in the PICOS format: population of interest (P), participants suffering from sudden onset of ISSNHL; intervention (I), participants receiving intratympanic steroids as initial treatment; comparison (C), participants receiving oral/systemic steroids as initial treatment; outcome (O), hearing recovery as well as adverse events; study design (S), RCT.^[31]^

All potentially relevant RCTs published up to December 2016 were systematically identified by searching Medline on OvidSP, PubMed, Embase, CINAHL, and the Cochrane Library. An additional search of the gray literature was performed accessing Google Scholar. Furthermore, hand searches of the references from the included studies were performed. The search strategy was similar for all databases and is exemplified here for the case of PubMed: (1) sudden hearing loss, (2) idiopathic sudden sensorineural hearing loss, (3) sudden sensorineural hearing loss, (4) sudden deafness, (5) (1–4)/OR, (6) systemic steroids, (7) oral steroids, (8) intratympanic, (9) (6–8)/OR, and (10) (5 AND 9).

### 2.2 Inclusion and exclusion criteria

This study was approved by the Ethics in Research Committee at West China Hospital of Sichuan University. Search results were limited to human studies published in English that reported the change in PTA thresholds and the proportion of patients showing improved hearing after treatment.^[29,32]^ These were the primary outcomes of the analysis, while secondary outcomes were word recognition score (WRS) and adverse events.

We excluded case studies, case series, qualitative studies, noncontrolled studies, editorials, and books. Duplicate publications and studies involving steroid therapies delivered in combination with other treatments were also excluded.

### 2.3 Study selection, data extraction, and quality assessment

Titles and abstracts of all retrieved citations were screened independently by 2 authors (DL and FZ) to identify potentially relevant studies. Discrepancies were resolved by discussion. The following data were extracted independently by the same 2 authors using a standard form: general information, trial design, participant baseline characteristics, interventions, outcomes, and conclusions.

The same 2 authors then independently assessed the methodological quality of the included RCTs. Discrepancies were resolved by discussion. Risk of bias was assessed using the recommendations in the Cochrane Handbook for Systematic Interventions (version 5.1.0, http://www.handbook.cochrane.org),^[33]^ which focuses on bias related to randomization sequence generation, allocation concealment, blinding of participants or healthcare providers, incompleteness, reporting, and other issues.

### 2.4 Statistics and data analysis

Rev Man 5.3 software was used for all statistical analysis, with a significance threshold of *P* < .05. Dichotomous outcomes were analyzed by calculating odds ratios (ORs) and the corresponding 95% confidence intervals (CIs). Continuous outcomes were analyzed by calculating the mean difference and 95% CI. The type of meta-analysis model depended on whether significant heterogeneity was present in the pooled data. If he I^2^ value was <0.5, the fixed model was used; otherwise, the random model was used. Heterogeneity was assessed using the chi-squared and I^2^ tests, with I^2^ > 50% considered as significant heterogeneity.^[34]^ In cases of heterogeneity, potential causes were analyzed. Sensitivity analysis was also performed to assess the stability of results.

## 3 Results

### 3.1 Literature search

**Figure 1:**
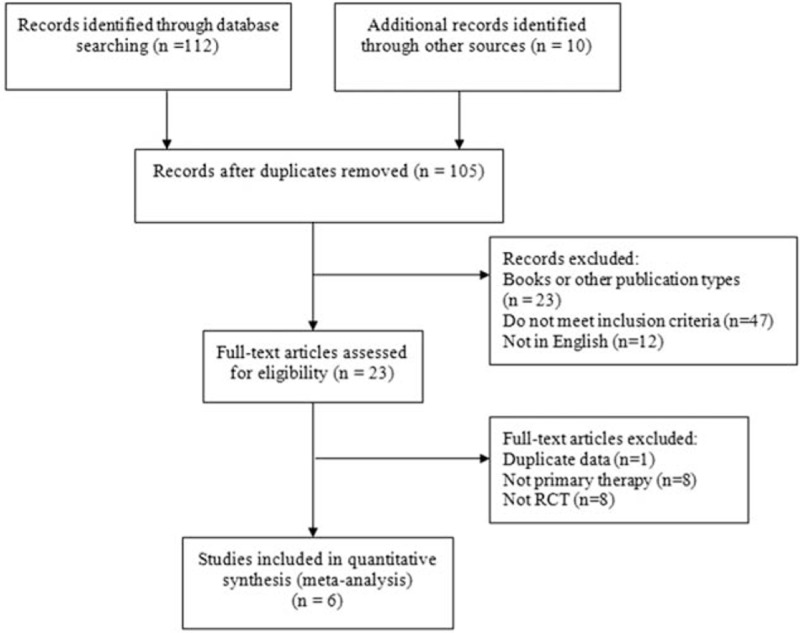
Flow diagram of study selection.

Literature searching initially turned up 105 potentially relevant studies (Fig. 1), of which 23 were read in full. Intratympanic steroid therapy was the primary treatment in 15 studies, while oral/systemic steroid therapy was the treatment in the remaining studies. In the end, 6 RCTs were included: 1 multicenter, double-blind study and 5 single-center prospective studies (Table 1).

**Table 1:**
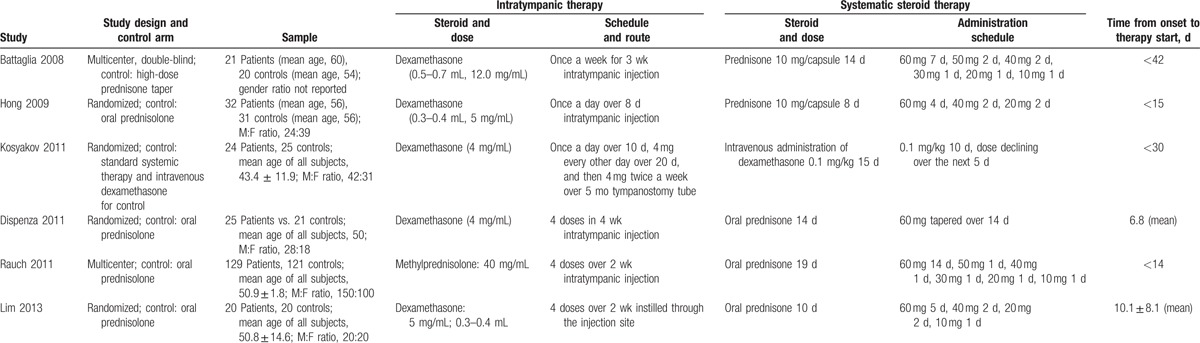
Characteristics of the included studies.

The 6 studies^[29,32,35–38]^ involved 484 participants with ages ranging from 20 to 83 years and a male:female ratio of 1.4:1. Intratympanic therapies involved either dexamethasone or methylprednisolone. Doses and duration of therapies varied widely across the studies; for example, total dexamethasone dose varied from 6^[36]^ to 240 mg,^[35]^ and duration of therapy varied from 8 days^[32]^ to 6 months.^[35]^

### 3.2 Comparative efficacy of intratympanic or systemic steroid therapy

Five studies reported no significant differences in average PTA improvement or recovery rate between the intratympanic and systemic treatment groups at 1 to 3 months after therapy initiation (*P* > .05).^[29,32,35,36,38]^ Three studies assessed hearing outcomes at 6 months: 2 of them found no significant difference,^[37,38]^ while 1 study found intratympanic therapy to be associated with greater hearing recovery (*P* < .05).^[35]^ Studies varied substantially in how they calculated PTA thresholds, how they defined hearing recovery, and when they assessed recovery for the last time. For example, 1 study calculated PTA using 3 low- and mid-frequencies,^[29]^ while the remaining 5 studies used 4 frequencies. The final assessment time point varied between 21 days^[36]^ and 6 months^[35,37,38]^ among the 6 studies. The outcomes assessment was shown in Table 2.

**Table 2:**
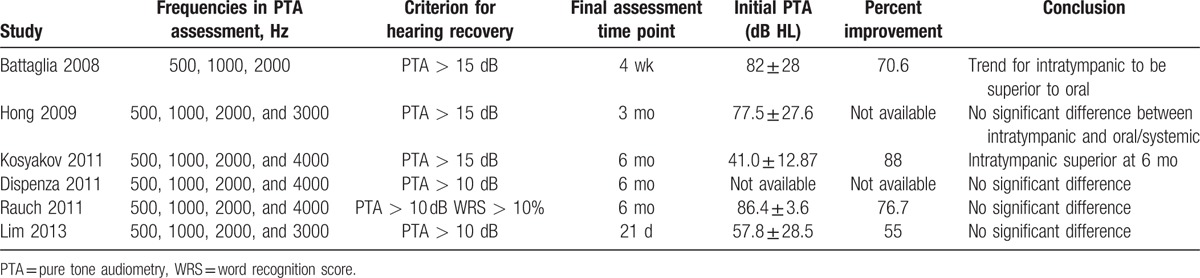
Outcome assessment.

**Figure 2:**
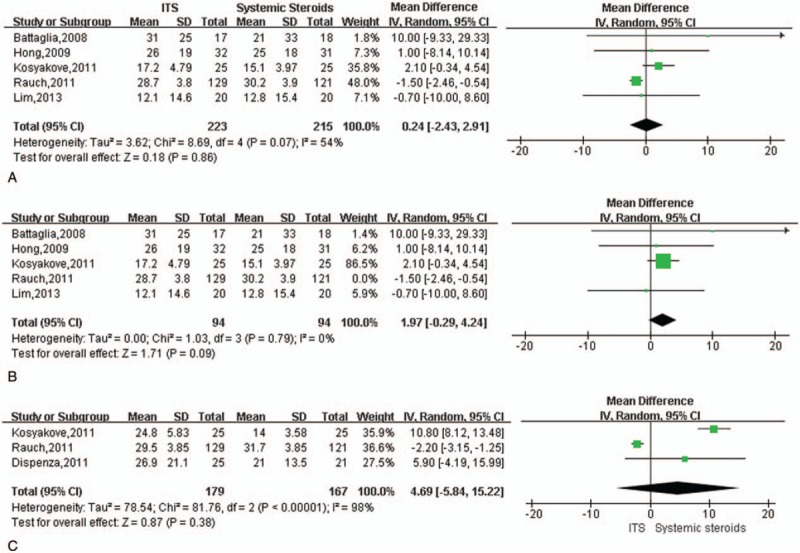
Meta-analysis of PTA improvement during intratympanic or oral/systemic steroid therapy to treat ISSNHL. (A) Forest plot of PTA improvement at 1 to 3 months after initiation of therapy. (B) Sensitivity analysis of the PTA improvement meta-analysis, showing that the study by Rauch 2011 provides the strongest evidence for equivalence of the 2 types of therapy. (C) Forest plot of the proportion of patients showing hearing recovery based on PTA improvement against a defined threshold. ISSNHL = idiopathic sudden sensorineural hearing loss, PTA = pure tone average.

Meta-analysis was performed on outcome assessments at 1 to 3 months in 5 trials.^[29,32,35,36,38]^ No significant difference in PTA improvement was observed between intratympanic and systemic therapy (mean difference, 0.24, 95% CI −2.43 to 2.91, *P* = .86; I^2^ = 54%, *P* = .07; random-effects model; Fig. 2A). Subgroup analysis was not performed because of the small number of trials and the heterogeneity in dose and duration of intratympanic therapy.

Sensitivity analysis indicated that the sole trial of Rauch et al,^[38]^ which accounted for more than half the patients in the meta-analysis, was the most powerful evidence for equivalence between intratympanic and systemic therapy (Fig. 2B). Among the 3 studies reporting outcome assessment at 6 months,^[35,37,38]^ meta-analysis of pooled data showed similar PTA thresholds (mean difference, 4.69; 95% CI −5.84 to 15.22, *P* = .38) with significant heterogeneity (I^2^ = 98%; Fig. 2C).

**Figure 3:**
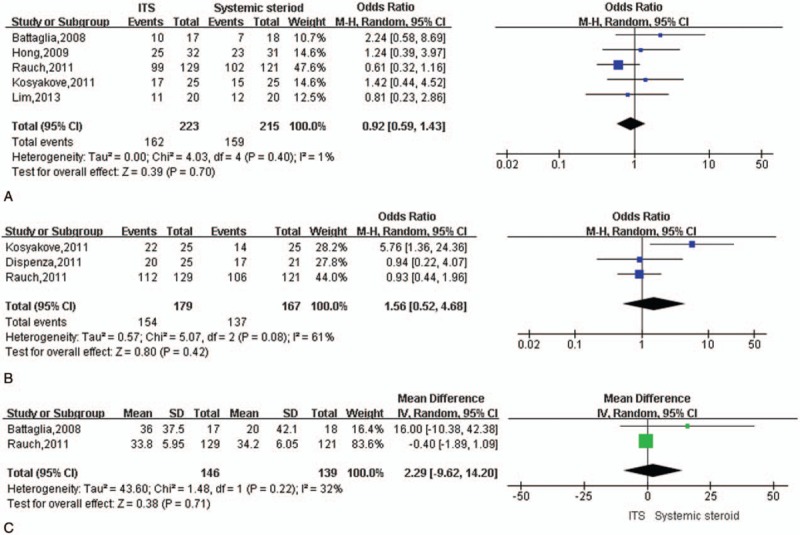
Meta-analysis of hearing recovery rate during intratympanic or oral/systemic steroid therapy to treat ISSNHL. (A) Forest plot of hearing recovery rate at 1 to 3 months after initiation of therapy. (B) Forest plot of hearing recovery rate at 6 months after initiation of therapy. (C) Forest plot of WRS at 1 to 2 months after initiation of therapy. ISSNHL = idiopathic sudden sensorineural hearing loss, WRS = word recognition score.

As another primary outcome, the rate of hearing recovery was compared between intratympanic and systemic steroid therapy. Three studies defined hearing recovery as an improved in PTA threshold >10 dB^[36–38]^; the other 3 studies defined it as an improved >15 dB.^[29,32,35]^ Data pooled from 5 studies^[29,32,35,36,38]^ showed similar hearing recovery rates between the 2 types of treatment at 1 to 3 months (OR 0.92, 95% CI 0.59–1.43, *P* = .70) without significant heterogeneity among studies (I^2^ = 1%, *P* = .40; Fig. 3A). Similar results were observed at 6 months (OR 1.56, 95% CI 0.52–4.68, *P* = .42), although heterogeneity was significant (I^2^ = 61%, *P* = .08; Fig. 3B). Sensitivity analysis identified the study by Kosyakov et al^[35]^ as the cause of the heterogeneity.

Two studies compared therapeutic efficacy in terms of WRS at 1 to 2 months.^[29,38]^ Meta-analysis of data pooled from these studies revealed no significant difference between the 2 types of treatment (mean difference, 2.29; 95% CI −9.62 to 14.2, *P* = .42) with no significant heterogeneity (I^2^ = 32%, *P* = .22; Fig. 3C).

### 3.3 Risk of bias

**Figure 4:**
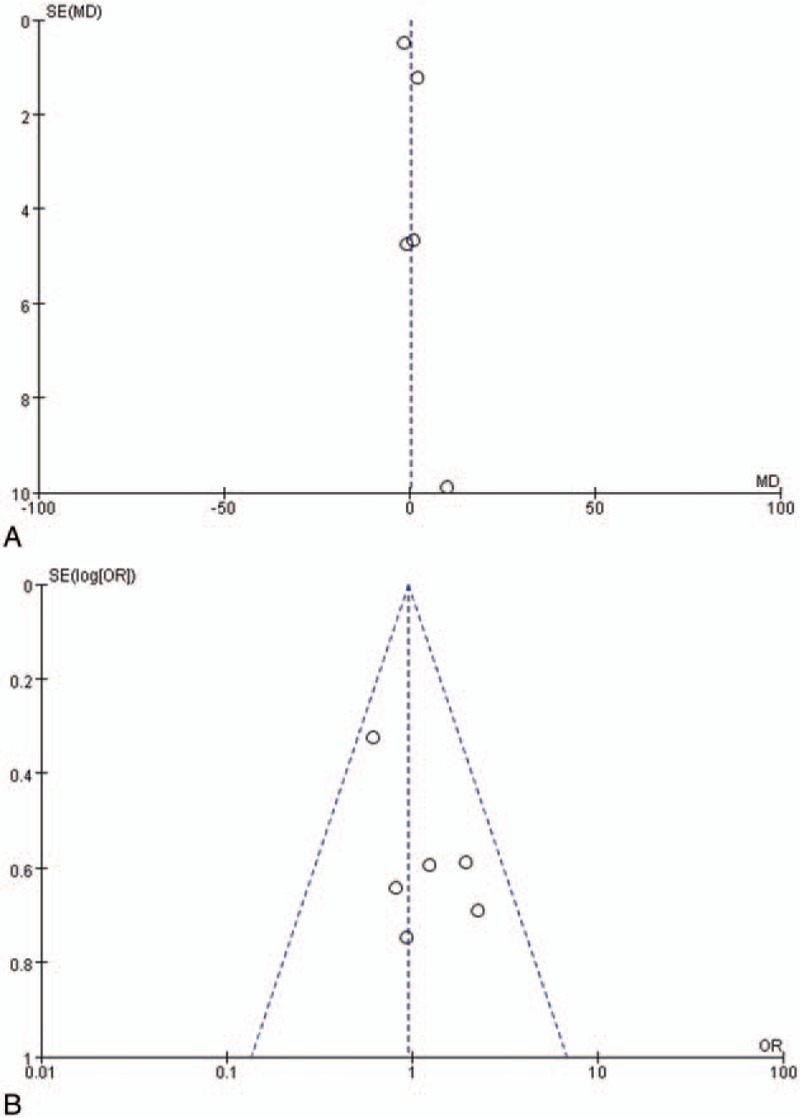
Meta-analysis of funnel plot of pure tone average improvement (A) and recovery rate (B) of comparing intratympanic steroid with systemic steroid therapy.

Despite being prospective RCTs, some studies showed high or unclear risk of selection and performance bias (Table 3). For example, the study by Rauch et al^[38]^ was a multicenter, prospective, randomized, noninferiority controlled trial with clearly detailed randomization and concealment procedures. However, participants and physicians were not blinded to treatment, increasing risk of performance bias. The other 5 studies^[29,32,35–37]^ had a similar blinding issue, and they also failed to describe in detail the randomization and allocation methods. Potential bias in meta-analysis was evaluated by funnel plots, as shown in Fig. 4, which indicated no obvious publication bias in our results.

**Table 3:**
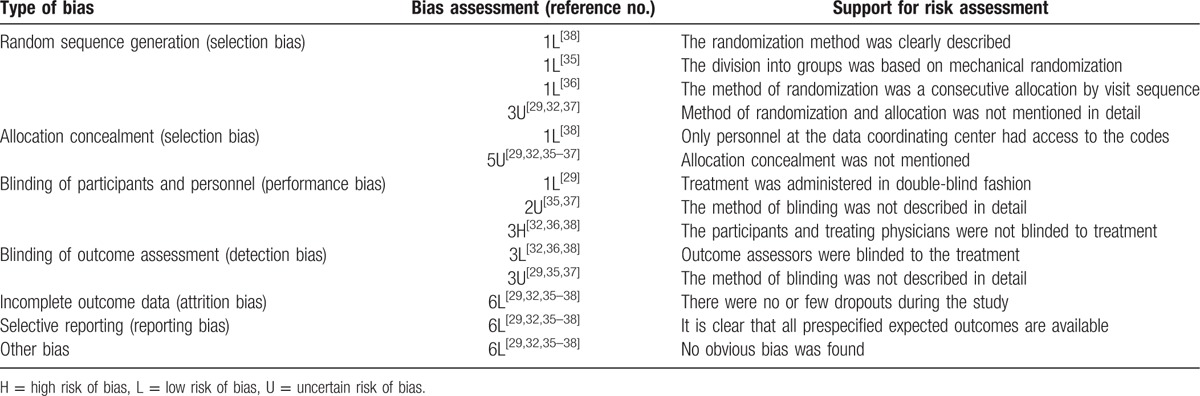
Risk of bias assessment.

### 3.4 Comparison of adverse effects

Adverse effects were reported in 1 study,^[38]^ 4 studies reported that no side effects were observed,^[29,32,36,37]^ and 1 study did not mention whether side effects occurred or not^[35]^ (Table 4). In the study reporting adverse events, none of the events was serious. The major events in the intratympanic group were ear pain, transient vertigo, ear infection, and persistent tympanic membrane perforation. No systemic adverse events were observed in this group. The major events in the oral group were a decrease in blood glucose, changes in sleep or appetite, dry mouth/thirst and weight change; all these events occurred at significantly higher frequencies than in the intratympanic group. Similar proportions of patients in each treatment group suffered ear infection.

**Table 4:**
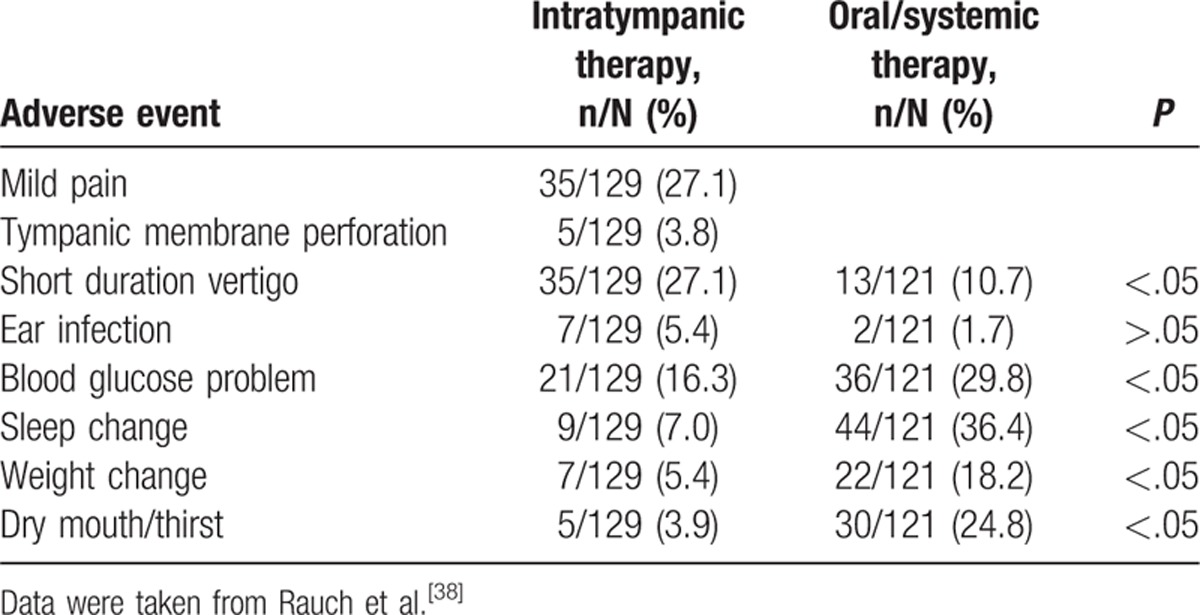
Adverse events associated with intratympanic or oral/systemic steroid therapy.

## 4 Discussion

Previous meta-analyses comparing intratympanic and oral/systemic steroid therapy as first-line treatment for ISSNHL have not resolved the controversy over which therapy is superior, probably in part because they included non-RCTs and heterogeneous treatment regimes in which intratympanic therapy was sometimes combined with other regimes. For example, 3 meta-analyses assessed only intratympanic steroid therapy used on its own,^[18,27,28]^ while 3 others included intratympanic therapy combined with systemic therapy.^[39–41]^

Here, we meta-analyzed only RCTs that compared intratympanic therapy on its own with oral/systemic therapy on its own. We found that the 2 therapies gave similar hearing recovery at 1 to 3 months and potentially also at 6 months after initiation of treatment. Recovery was measured in terms of PTA thresholds and the proportion of patients meeting a defined recovery threshold. Our finding contrasts with an earlier systematic review^[26]^ that also included only RCTs but did not include the large trial by Rauch et al.^[38]^ This large trial was, in our meta-analysis, the strongest evidence for equivalence between the 2 types of therapy.

Dexamethasone appears to be the steroid most frequently used to treat ISSNHL. This synthetic corticosteroid is widely used as an anti-inflammatory agent delivered orally, parenterally, and topically. Studies suggest that dexamethasone is effective against ISSNHL because it decreases inflammation from labyrinthitis,^[42]^ improves cochlear blood flow,^[43]^ protects against cochlear ischemia,^[44]^ and improves striavascularis function. It may exert these effects by modulating Na^+^/K^+^ ion exchange, helping to maintain endocochlear potential and thereby promoting hearing recovery.^[45]^ In addition to dexamethasone, methylprednisolone is recommended to treat ISSNHL.^[38,46,47]^ Pharmacokinetics studies show that it accumulates primarily in the inner ear.^[12]^

The 6 studies in this meta-analysis showed substantial variation in the dose and duration of intratympanic steroid delivery. For example, total doses of intratympanic dexamethasone varied from 6 to 240 mg delivered over 8 days to 6 months. This highlights the need for greater standardization of intratympanic therapy.

Studies also differed substantially in how researchers analyzed improvement in PTA thresholds, with each study using a different frequency range. Some studies defined hearing recovery as a PTA increase >10^[36–38]^ or >15 dB.^[29,32,35]^ These observations highlight the need for greater standardization of hearing assessments during treatment of patients with ISSNHL. We suggest that future studies should aim to standardize the frequency range for PTA and should define normal variability in PTA thresholds as 5 to 10 dB,^[48]^ which argues for using >10 dB as the definition of hearing recovery.^[1]^ Hearing assessments during treatment for ISSNHL should also include calculation of the WRS,^[1]^ which was performed in only 2 trials.^[29,38]^

Another aspect of intratympanic therapy yet to be standardized is the delivery system. In most studies, the steroid was delivered by transtympanic injection,^[29,32,37,38]^ which can be less invasive, safer, and more cost-effective than other methods. On the other hand, it can lead to early, uncontrollable loss of the steroid solution through the Eustachian tube.^[49]^ Another possibility is instillation of the steroid solution through a ventilation tube in the tympanic membrane,^[35]^ in which case the patient can administer the drug himself or herself. However, how much of the applied solution enters the inner ear is uncertain. Future studies should rigorously compare the various intratympanic delivery systems.

Finally, the present review makes clear a striking lack of safety assessment in studies of primary treatments for ISSNHL. Few studies in our review reported such data, despite being RCTs. While the available evidence hints that intratympanic therapy may be preferable to systemic therapy, more data of higher quality are needed in order to draw definitive conclusions. Future research should comprehensively examine the adverse events and side effects of intratympanic therapy.

## 5 Conclusion and future research

This review and meta-analysis of English-language RCTs found no evidence of significant difference in short-term hearing recovery obtained with intratympanic or oral/systemic steroid therapy for patients with ISSNHL. This supports the use of intratympanic therapy over systemic therapy when avoiding potential steroid side effects is more important, such as for patients with other chronic diseases. Review of the RCTs here indicates large gaps in our understanding of adverse events and long-term efficacy of intratympanic steroid therapy; it also highlights the need for standardized procedures for PTA-based assessment of hearing recovery.
